# Identification of Candidate Genes Regulating the Seed Coat Color Trait in Sesame (*Sesamum indicum* L.) Using an Integrated Approach of QTL Mapping and Transcriptome Analysis

**DOI:** 10.3389/fgene.2021.700469

**Published:** 2021-08-04

**Authors:** Chun Li, Yinghui Duan, Hongmei Miao, Ming Ju, Libin Wei, Haiyang Zhang

**Affiliations:** ^1^Henan Sesame Research Center, Henan Academy of Agricultural Sciences, Zhengzhou, China; ^2^Henan Key Laboratory of Specific Oilseed Crops Genomics, Henan Sesame Research Center, Henan Academy of Agricultural Sciences, Zhengzhou, China

**Keywords:** seed coat color, sesame, genetic linkage map, genome re-sequencing, RNA-Seq

## Abstract

Seed coat color is an important seed quality trait in sesame. However, the genetic mechanism of seed coat color variation remains elusive in sesame. We conducted a QTL mapping of the seed coat color trait in sesame using an F_2_ mapping population. With the aid of the newly constructed superdense genetic linkage map comprised of 22,375 bins distributed in 13 linkage groups (LGs), 17 QTLs of the three indices (i.e., *L*, *a*, and *b* values) of seed coat color were detected in seven intervals on four LGs, with a phenotype variance explanation rate of 4.46–41.53%. A new QTL *qSCa6.1* on LG 6 and a QTL hotspot containing at least four QTLs on LG 9 were further identified. Variants screening of the target intervals showed that there were 84 genes which possessed the variants that were high-impact and co-segregating with the seed coat color trait. Meanwhile, we performed the transcriptome comparison of the developing seeds of a white- and a black-seeded variety, and found that the differentially expressed genes were significantly enriched in 37 pathways, including three pigment biosynthesis related pathways. Integration of variants screening and transcriptome comparison results suggested that 28 candidate genes probably participated in the regulation of the seed coat color in sesame; of which, 10 genes had been proved or suggested to be involved in pigments biosynthesis or accumulation during seed formation. The findings gave the basis for the mechanism of seed coat color regulation in sesame, and exhibited the effects of the integrated approach of genome resequencing and transcriptome analysis on the genetics analysis of the complex traits.

## Introduction

Sesame (*Sesamum indicum* L.) is a specific and important oilseed crop with a long cultivation history (Bedigian, 2003). At present, sesame is mainly grown in Asia, Africa, and Latin America. Sesame seeds possess abundant nutritional substances, such as unsaturated fatty acids, proteins, digestible fiber, and beneficial antioxidants (such as lignans) (Namiki, 2007; Kumar et al., 2013; Ha et al., 2017; Keskin, 2019). For sesame seeds, seed coat color varies from white, shallow yellow, yellow, golden, brown, gray, reddish brown, and other medium colors, to black. In particular, the white- and the black-seeded seeds are more popular for consumption in the world. Black sesame seeds are shown to possess abundant nutritional facts (Ganesan and Xu, 2017; Khoo et al., 2017). Some studies showed that the contents of some substances, such as phenolics, vitamins, and phloretin, in black sesame seeds are high (Ha et al., 2017; Wang et al., 2018). In China, black sesame seeds have also been used as a traditional Chinese medicine in the last 3,000 years (Wang et al., 2018; Zhang H. et al., 2019).

In sesame, seed coat color trait is considered as a quantitative trait (Zhang H. et al., 2013; Wang L. et al., 2016). Zhang H. et al. (2013) firstly performed the genetic background and QTL mapping analysis of sesame seed coat color with an F_2_ hybridization population using simple sequence repeat (SSR), amplified fragment length polymorphism (AFLP), and random selective amplification of microsatellite polymorphic loci (RSAMPL) markers. Four QTLs were located on three LGs with the phenotypic variation from 9.6 to 39.95% (Zhang H. et al., 2013). Subsequently, Wang L. et al. (2016) investigated the seed coat color indices of the *L*, *a*, and *b* values of a recombinant inbred lines (RIL) population and determined four QTLs on a genetic map constructed by restriction-site associated DNA sequencing (RAD-Seq). The four QTLs were found to be on three LGs and with the phenotypic variation from 3 to 46%. Wei et al. (2015) performed a genome-wide association study (GWAS) for the seed coat color and some other traits using a panel of 705 sesame accessions and determined seven loci associated with the seed coat color. Meanwhile, Du et al. (2019) detected seven QTLs related with seed coat color on three LGs using specific length amplified fragment sequencing (SLAF-seq) and an F_2_ population. Wang et al. (2020) identified 20 candidate genes associated with pigment biosynthesis using transcriptome data from a black and white sesame variety. However, only the *SiPPO* (polyphenol oxidase) gene was determined for the seed coat color in sesame until now (Wei et al., 2015).

For angiosperm plants, the seed coat color trait is predominantly determined by the content of various pigments, which mainly belong to the secondary flavonoid metabolites, such as flavonols, anthocyanins, and proanthocyanidins (PAs), and are synthesized through the flavonoid biosynthesis pathway (Winkel-Shirley, 2001). At present, dozens of genes regulating the seed coat color regulation have been detected in crops; of which, most genes encode the enzymes related to flavonoid biosynthesis (such as *C2* in maize, and *TT6* and *TT7* in *Arabidopsis*), transfer proteins (*ZmMRP3* in maize and *TT12* and *AHA10* in *Arabidopsis*), or regulatory factors (*TT1* and *TT10* in *Arabidopsis*) (Pourcel et al., 2005; Marinova et al., 2007; Owens et al., 2008; Badone et al., 2010; Han et al., 2010; Appelhagen et al., 2011, 2015; Eloy et al., 2017). Flavonoid biosynthesis pathway is highly conserved in different plant species (Dong et al., 2001; Tohge et al., 2017). To date, numerous transcription factors, such as MYBs, basic helix-loop-helix (bHLHs), Transparent Testa Glabra1 (*TTG1*), and MYB-bHLH-WDR (MBW) ternary complexes, have been found to be participating in the regulation of flavonoid biosynthesis pathway in a tissue-specific manner (Gonzalez et al., 2009; Xu et al., 2014). In addition, post-translation modification and microRNA have also been proven to be related to the flavonoid biosynthesis pathway (Wang H. et al., 2016).

In this study, in order to identify the major effect of genes underlying sesame seed coat color, we performed a QTL mapping for the sesame seed coat color trait based on a superdense genetic linkage map constructed using an F_2_ population and genome resequencing technology. The transcriptome comparison analysis of a black- and white-seeded variety during seed development was also carried out. As a result, a total of 28 candidate genes were determined for the seed coat color trait in sesame according to the integration of QTL mapping and transcriptome analysis. The findings provide a deep understanding of the genetic mechanism underlying the seed coat color regulation in sesame.

## Materials and Methods

### Materials and Data

Two varieties, Yuzhi DS899 (white-seeded, mutated from a sibling of Yuzhi 11) and JS012 (black-seeded), and their 120 F_2_ individuals were used for the QTL mapping in this study (Zhang et al., 2016). The F_2_ population and both parents were grown in Sanya (109°50′E and 18°25′N), Hainan, China in 2014 for seed coat color trait evaluation. For the transcriptome analysis, two varieties with a contrasting seed coat color, i.e., Yuzhi 11 and the black-seeded parent JS012, were cultivated in Yuanyang (113°97′E and 35°05′N), China in 2020. Twenty capsules from 10 plantlets of each variety at the time points 5, 10, 15, 20, and 25 DAF, respectively, were collected. The developing seeds were then peeled from the capsules, frozen in liquid nitrogen, and stored at –80°C for RNA extraction. Three biological replicates were set for RNA sequencing. All the materials above were chosen from the sesame germplasm reservoir of Henan Sesame Research Center, Henan Academy of Agricultural Sciences (HSRC, HAAS).

The genome resequencing data (reserved in NCBI Bioproject PRJNA315474) of Yuzhi DS899 and JS012 and their 120 F_2_ individuals (Zhang et al., 2016) were used for the genetic map construction and QTL mapping in this study. The sesame genome of v*ar.* Yuzhi11 (white-seeded; version 2) (Zhang et al., 2016) was used as the reference genome for genotyping, variant digging, and candidate gene screening analysis.

### Seed Coat Color Trait Evaluation

Seed coat color of the F_2_ family was investigated based on a single plant. For each of the sequenced 120 F_2_ individuals above, the seeds were harvested after the plant was totally matured. The seeds from each individual were randomly divided into three equal pieces (used as three replicates) after the unmatured seeds were discarded. As to evaluate the seed coat color, the seeds were put into a quartz cell, and then scanned under the Colorflex EZ spectrophotometer (Hunter Associates Laboratory, United States). Three indices (i.e., *L*, *a*, and *b* values) were measured and recorded. Of which, “*L*” defines the luminosity of the seed coat, with a range from –100 for black to 100 for white; “*a*” and “*b*” indicate the shade of color pairs, with a range from –60 for green to +60 for red, and from –60 for blue to +60 for yellow, respectively. For each of the indices, the mean value of the three replicates was finally used.

### Construction of a Superdense Genetic Linkage Map for Sesame

Sequencing reads of the parents Yuzhi DS899 and JS012 and their 120 F_2_ individuals (Zhang et al., 2016) were firstly filtered using the Trimmomatic 0.33 software (Bolger et al., 2014), and then mapped to the reference sesame genome (*cv.* Yuzhi 11) (Zhang et al., 2016) using the Burrows Wheeler Aligner (BWA) 0.7.17 software with the default settings (Li and Durbin, 2009). Joint variant calling was performed using the GATK 4.0 program following its best practice (Van der Auwera et al., 2013). Genotype coding was performed using the GenosToABH plugin in TASSEL 5.2.43 (Glaubitz et al., 2014). The homozygous genotype of the parent Yuzhi DS899 was coded as “A,” the parent JS012 as “B,” and the heterozygous genotype of their hybrid as “H.” Since several consecutive heterozygotes (“H”) would be called as identical homozygotes (“A” or “B”) within a very short distance due to the low reads coverage, the coded genotype was processed according to Miao et al. (2018) using the following procedures to improve the accuracy of linkage map construction: (1) combining the consecutive and redundant (with identical information for all individuals in the progeny) markers within a short region into a representative marker, and the minimum length between two continuous markers was set to 100 bp (base pair); (2) correcting the genotype of representative markers based on a sliding window of 15 markers; (3) based on the corrected markers, grouping the adjacent and redundant markers into bins; and (4) filtering out the bins of distorted segregation with the number of plants with “A” or “B” genotypes <20, and the number of plants with H genotypes <40. The threshold for distorted segregation loci was selected based on a pilot study of linkage map construction, in which we found that at least 80 individuals (with ideal segregation ratio 1:2:1 for genotype “A,” “B,” and “H”) were needed to form 13 linkage groups (corresponding 13 chromosomes of sesame).

The genetic map was constructed based on the filtered genotype matrix using the Lep-MAP3 program with the default parameters and a logarithm of the odds (LOD) of 12.0 to separate chromosomes (Rastas, 2017). LGs with less than 100 markers were discarded. The Kosambi function was applied to transfer the recombinant rate into the genetic distance (Morgan).

### QTL Mapping and Comparison

QTL mapping was performed using the QTL Cartographer 2.5 (Wang et al., 2012), QTL.gCIMapping.GUI v1.0 (Wen et al., 2019; Zhang Y. W. et al., 2019), and QTL IciMapping 4.2.53 (Meng et al., 2015), respectively. The Composite interval mapping (CIM) method in the QTL Cartographer, the Genome-wide composite interval mapping (GCIM) method in the QTL.gCIMapping.GUI, and the Inclusive composite interval mapping (ICIM) and Interval mapping (IM) methods in the QTL IciMapping were individually used to detect the QTLs and to estimate the effects. For the CIM method, the map was scanned in 2 cM (centimorgan) intervals with a window size of 10 cM, and the LOD was determined by running 1,000 permutation tests. For the GCIM method, random model, critical LOD score = 2.5, and walk step for genome wide scanning = 1 cM were used. For the ICIM and IM methods, LOD was determined by running 1,000 permutation tests at type I error equals 0.05, and walk step was set at 1 cM. The ratio of phenotypic variance explained by genotype was taken from the marker at the peak QTL position. QTL physical position was determined by the flanking markers.

To make a distinction between the new QTLs identified in this study and from the reported results, QTL position comparison was performed with flanking sequences using the MUMER 4 program (Marcais et al., 2018). The flanking sequences of the QTLs or the associated loci were applied as the queries for alignment against the sesame reference genome (*cv.* Yuzhi 11) (Zhang et al., 2016).

### RNA-Seq and Analysis

Total RNA of each sample was extracted using the RNAiso Plus Reagents (TaKaRa, Dalian, China). Genomic DNA was removed by DNase I treatment. RNA-seq libraries were constructed and sequenced on the Illumina platform HiSeq 2500 (Illumina, Inc., San Diego, CA, United States). The clean reads were mapped to the reference sesame genome using the STAR 2.7.0 (Dobin et al., 2013). The reads mapped to each genomic feature were counted using the featureCounts 2.0.1 (Yang et al., 2014). Default parameters were used for both read mapping with STAR and read counting with featureCounts. Differentially expressed genes (DEG) were screened and analyzed using the DESeq 2 software with the default parameters (Love et al., 2014). False discovery rate (FDR) adjusted *p*-values < 0.05 and absolute value of log_2_ (fold change) > 1 were set as the cutoff for the DEGs.

### Gene Function Annotation, Homologous Gene Screening, and Variant Effect Prediction

Gene function annotation was performed using the eggNOG-Mapper 5.0.1 (Huerta-Cepas et al., 2017). Gene ontology (GO) plotting was conducted in the WEGO 2.0 (Ye et al., 2018). Homologous gene screening in the target intervals was conducted using the BLAST + 2.2.31 with the *E*-value < 1e-10. The effect of gene variant was estimated using the SnpEff 4.1 (Cingolani et al., 2012), and the variants that caused frame shift, stop gain, stop lost, start lost, splice acceptor, splice donor, exon loss, frame-shift, or nonsense mutation were regarded as high-impact and screened according to Cingolani et al. (2012). Kyoto Encyclopedia of Genes and Genomes (KEGG) pathway enrichment analysis was performed using the R package clusterProfiler 3.12 with the default parameters (Yu et al., 2012), and a *q*-value < 0.05 was set as the cutoff.

## Results

### Seed Coat Color Genetic Analysis of the Mapping Population

In order to clarify the genetic background of the seed coat color in sesame, we firstly investigated the phenotypic variation of the seed coat color trait of the F_2_ population in 2016 (Supplementary Table 1 and Supplementary Figure 1). *L*, *a*, and *b* values of the white-seeded parent Yuzhi DS899 were 62.81, 5.50, and 19.62, respectively, while those of the black seeded parent were 21.62, 6.73, and 7.27, respectively. All the F_1_ seeds were black, whereas F_2_ plants exhibited an obvious variation in seed coat color with various values of *L*, *a*, and *b* (Supplementary Figure 1). As to the 120 F_2_ plants, “*L*” values ranged from 16.23 to 62.81, with a standard deviation of 8.40; the “*a*” values ranged from 1.20 to 9.34, with a standard deviation of 2.67; while “*b*” varied from 1.42 to 19.62, with a standard deviation of 4.27 (Supplementary Table 1). In particular, we found some F_2_ plants that presented a darker seed coat color than the black parent (Supplementary Figure 1). However, the color of all the F_2_ plantlets was darker than the white-seeded parent.

**FIGURE 1:**
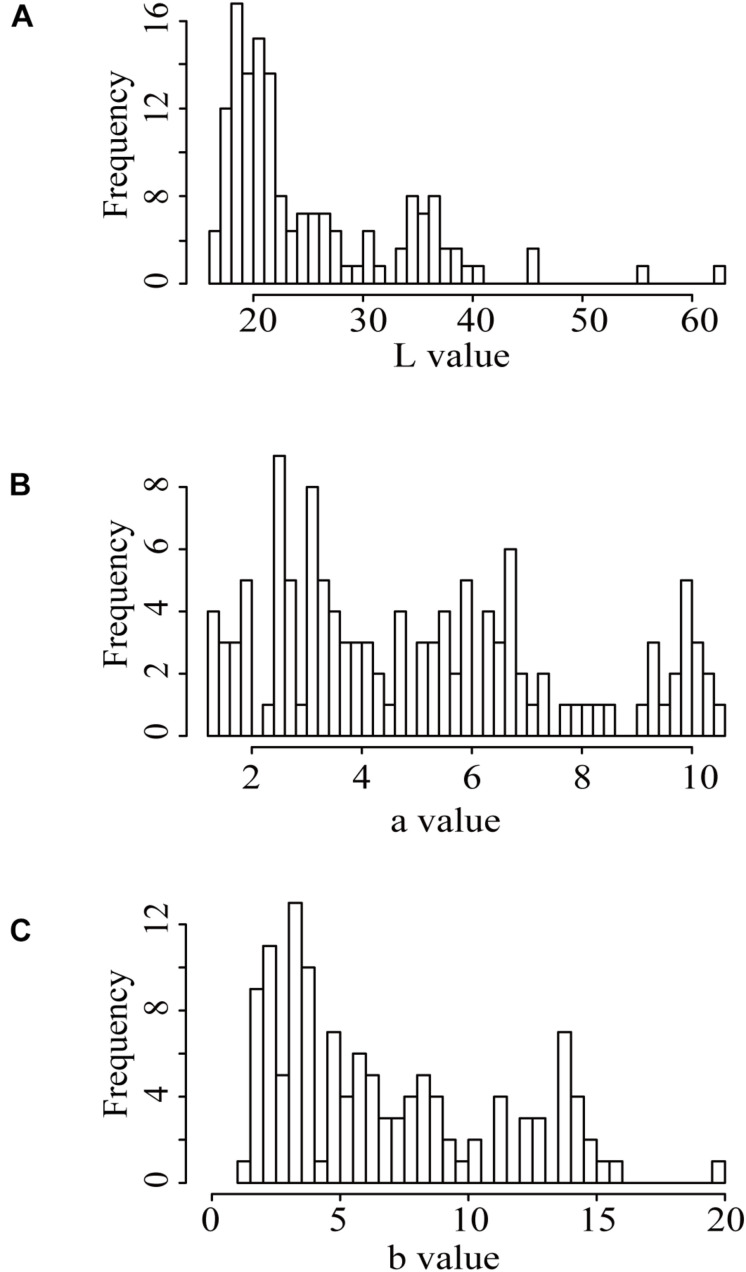
Distribution of the seed coat color indices presented by the *L*, *a*, and *b* values in the F_2_ population. **(A)** “*L*” value; **(B)** “*a*” value; **(C)** “*b*” value.

We further performed the pairwise correlation analysis of the three indicators of the seed coat color in the population (Supplementary Table 1). The results showed that the *L*, *a*, and *b* values were significantly correlated with each other. The *L*, *a*, and *b* values of the F_2_ population were all out of the normal distribution (Figure 1 and Supplementary Table 1), indicating the existences of a few major QTLs controlling the three indicators.

### Construction of a Superdense Genetic Linkage Map

Before determining the QTLs for the seed coat color trait, we firstly constructed a superdense genetic map using the F_2_ population above and the genome resequencing data. Based on the reference genome (*var.* Yuzhi 11), a total of 1,264,646 SNP/InDel variants for the two parents and their 120 F_2_ plants were identified (Supplementary Table 2). The number of unique variants for the two parents Yuzhi DS899 and JS012 was 122,361 and 528,671, respectively. After filtered, a total of 425,011 variants were successfully genotyped into “A,” “B,” or “H” types in the population (Supplementary Table 2).

**FIGURE 2:**
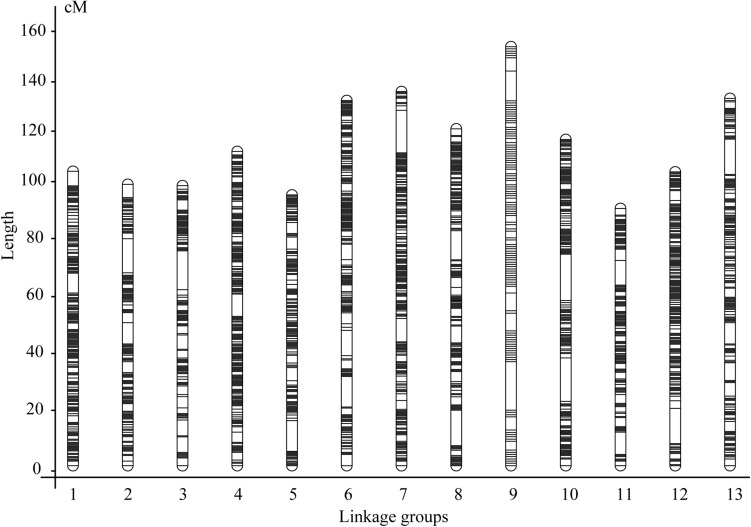
The superdense genetic map constructed using an improved pipeline and the F_2_ population derived from a cross between Yuzhi DS899 and JS012.

Then, we constructed the matrix consisting of the above 425,011 genotype codes for the 120 F_2_ individuals and fed it to the linkage map construction pipeline (Table 1). As the bins with the distorted segregation (the numbers of plants having A/B genotypes < 20 and H genotypes < 40) were excluded, a total of 22,375 bins containing 380,544 SNP/InDel markers were obtained for the genetic map construction (Table 1 and Supplementary Table 3). As a result, a genetic map with 13 LGs was successfully constructed (Figure 2). The total length of the genetic map was 1,576.14 cM, and the length per LG ranged from 96.96 to 157.96 cM. The average marker interval was 0.82 cM per bin marker. The marker density was 14.20 bins or 241.44 SNP/InDel markers per cM. In addition, 34 gaps with the length ≥ 5.0 cM remained in the genetic map, with the largest gap size of 18.27 cM (Table 1).

**TABLE 1:** Characteristics of the superdense genetic linkage map for sesame.

**LG**	**Length (cM)**	**Bin number**	**Variants number**	**Average interval (cM)**	**Gap number^1^**	**Largest gap (cM)**
1	110.96	1,512	27,577	0.66	2	7.56
2	106.05	1,823	28,880	0.87	3	12.97
3	105.61	981	20,903	1.03	4	14.57
4	118.46	2,234	37,066	0.63	1	8.41
5	102.08	1,339	18,558	0.77	2	11.60
6	137.67	1,794	26,621	0.81	3	11.47
7	141.10	3,090	49,745	0.73	2	15.97
8	126.98	1,930	32,522	0.86	3	13.34
9	157.96	1,705	24,462	1.36	5	18.27
10	122.99	1,383	29,655	0.8	2	17.36
11	96.97	1,379	25,062	0.81	2	9.30
12	110.80	1,835	30,175	0.67	1	13.27
13	138.52	1,370	29,318	0.89	4	13.27
Total	1,576.15	22,375	380,544	0.82	34	18.27

*^1^Only the marker interval with the size of >5.0 cM was considered as a gap.*

### QTL Mapping of the Seed Coat Color Trait in the F_2_ Population

We then performed a QTL mapping of the seed coat color in the crossing population using four methods (GCIM, ICIM, IM, and CIM) (Table 2 and Supplementary Table 4). The results from the four methods were similar, and QTLs were found on LG3, 5, 6, and 9 for the three seed coat color indicators *L*, *a*, and *b* (Table 2 and Supplementary Table 4). Of these, the QTL intervals observed using the CIM method were usually wider, while the intervals from the GCIM method were narrower. The overlapping relationship and boundaries between the QTLs were obvious (Supplementary Table 4). Therefore, based on the QTL interval boundaries, we summarized the QTL mapping resulting into 17 QTLs, including 5, 4, and 8 QTLs for *L*, *a*, and *b*, respectively (Table 2). The genetic effect (the explanation rate of phenotype variance or V_G_/V_P_ value) of the QTLs on the seed coat color trait ranged from 4.66% (for *qSCb5.2* estimated by GCIM) to 41.53% (for *qSCL9.3* estimated by ICIM).

**TABLE 2:** QTL distribution of the seed coat color using *L*, *a*, and *b* values in sesame.

**Trait**	**LG**	**QTL**	**Left marker**		**Right marker**		**VG/VP (%)^1^**	**Peak LOD^2^**	**Detected method**
			**Name**	**Pos (cM)**	**Name**	**Pos (cM)**			
*L*	3	*qSCL3.1*	C34-3876888	43.75	C34-3707103	47.09	6.58, 28.19	8.84, 15.00, 8.11	GCIM, ICIM, and CIM
	9	*qSCL9.1*	C3-4785376	9.17	C3-5518941	20.40	24.61,	13.82, 23.35	IM and CIM
		*qSCL9.2*	C3-5518941	20.40	C3-5518941	21.23	6.65	9.38	GCIM
		*qSCL9.3*	C3-5518941	21.23	C_2_3-7327811	27.07	39.42, 41.53, 20.57	38.07, 19.86, 24.11, 39.44	GCIM, ICIM, IM, and CIM
		*qSCL9.4*	C_2_3-7239571	28.73	C_2_3-6815285	30.40	19.39	19.36, 24.82	IM and CIM
*a*	5	*qSCa5.1*	C_2_7-2469423	20.44	C_2_7-2494612	22.11	7.00, 14.80	7.20, 7.49, 6.88	GCIM, ICIM, and CIM
	6	*qSCa6.1*	C4-7225533	78.80	U0167-32152	84.23	7.99, 11.92	6.44, 6.54, 7.01	GCIM, ICIM, and CIM
	9	*qSCa9.1*	C3-5590000	21.23	C_2_3-7327811	27.07	13.38, 31.97, 24.42	16.60, 15.19, 17.95, 18.45	GCIM, ICIM, IM, and CIM
		*qSCa9.2*	C_2_3-7239571	28.73	C_2_3-6815285	30.40	24.59	15.91, 16.08	IM and CIM
*b*	3	*qSCb3.1*	C34-3876888	43.75	C34-4228322	44.17	14.80	7.49	ICIM,
		*qSCb3.2*	C34-4201909	44.59	C34-4201909	44.59	4.73	5.16	GCIM
	5	*qSCb5.1*	C_2_7-2469423	20.44	C_2_7-2475883	21.27	11.96,	6.08	ICIM
		*qSCb5.2*	C_2_7-2476632	22.11	C_2_7-2476632	22.11	4.66	5.77	GCIM
	9	*qSCb9.1*	C3-4785376	9.17	C3-5518941	20.40	22.85,	10.47, 29.43	IM and CIM
		*qSCb9.2*	C3-5518941	20.40	C3-5518941	21.23	12.50	10.94	GCIM
		*qSCb9.3*	C3-5518941	21.23	C_2_3-7327811	27.07	31.58, 32.93, 19.33	29.97, 14.08, 22.60, 36.17	GCIM, ICIM, IM, and CIM
		*qSCb9.4*	C_2_3-7239571	28.73	C_2_3-6815285	30.40	18.00	17.76, 22.42	IM and CIM

*^1^The values from the CIM method were not included because the QTLs mapped on LG9 are clustered, CIM seems unable to distinguish the effect of each QTL. ^2^Peak LOD was calculated according to the GCIM, ICIM, IM, and CIM methods, respectively. The detail information is listed in Supplementary Table 4.*

Due to the overlapping positions of the QTLs controlling *L*, *a*, and *b*, a total of seven non-overlapping intervals, with one on LG3 (43.75–47.09 cM), one on LG5 (20.44–22.11 cM), one on LG6 (78.80–84.23 cM), and four on LG9 (9.17–20.4, 20.4–21.23, 21.23–27.07, and 28.73–30.40 cM), were identified for the sesame seed coat color in this study (Table 2). As the QTLs intervals on LG9 were adjacent to each other, we thus named the QTL hotspot “*qSC* cluster” for the seed coat color trait. By further merging this *qSC* cluster into one interval, a total of four intervals (hereinafter referred to as “*qSC* intervals”) on four LGs were thus harvested for the seed coat color trait in sesame, i.e., LG3: 43.75–47.09 cM, LG5: 20.44–22.11 cM, LG6: 78.80–84.23 cM, and LG9: 9.17–30.40 cM (Table 2).

### Genes in the qSC Intervals

We further screened the genes in the four *qSC* intervals according to the fine sesame reference genome (Table 3). The results showed that the above *qSC* intervals were located into six contigs (i.e., C34, C_2_7, C4, U0167, C3, and C_2_3) in the reference genome. The physical size of the six contig segments varied from 26.80 Kb (kilobases) (in contig U0167) to 2,090.72 Kb (in contig C_2_3). A total of 625 genes were detected in the six contig segments, and the number of genes in each of the contig segments varied from one on contig C4 to 235 on C_2_3 (Table 3 and Supplementary Table 5).

**TABLE 3:** Genes and variants in the four merged *qSC* intervals based on the sesame genome data.

**LG**	***qSC* Interval (cM)**	**Physical position (Kb)^1^**				**Gene number**	**SNP/InDel number^2^**	**Unique in JS012^3^**	
		**Located contig**	**Start**	**End**	**Size**			**Low-impact**	**High-impact**
3	43.75–47.09	C34	3,690.83	4,394.65	703.82	94	2,705	2,455	101
5	20.44–22.11	C_2_7	2,344.66	2,796.34	451.68	60	2,723	2,367	142
6	78.80–84.23	C4	7,225.53	7,261.46	35.93	1	7	7	0
		U0167	5.35	32.15	26.80	4	346	329	5
9	9.17–30.40	C3	4,285.99	6,348.88	2,062.88	231	695	163	3
		C_2_3	6,429.48	8,520.20	2,090.72	235	4,243	3,679	142
Total	–	–	–	–		625	10,719	9,000	393

*^1^The physical location of the QTL intervals was calculated based on the genome Yuzhi 11 (Zhang et al., 2016).*

*^2^SNP and InDel number is calculated based on the white- and black-seeded parents joint calling; “–” Not detected.*

*^3^Unique in JS012 (the black-seeded parent), comparing with Yuzhi DS899 (the white-seeded parent) and Yuzhi 11 (the variety used for reference genome sequencing, white-seeded). See Supplementary Table 5 for detail.*

In order to determine the candidate genes, we firstly compared the two parents and determined that a total of 10,719 SNP/InDel variants existed in the six target contig segments between the parents (Table 3 and Supplementary Table 6). Of which, 9,393 variants were unique in JS012 (black-seeded) compared with Yuzhi DS899 (white-seeded) and Yuzhi 11 (the variety used for reference genome sequencing, white-seeded), and thus, co-segregated with the seed coat color trait. Annotation results of SnpEff indicated that 393 variants in 84 genes caused frame shift, stop gain, stop lost, start lost, splice acceptor, splice donor, exon loss, frame-shift, or nonsense mutation, and were named as high-impact variants (Table 3 and Supplementary Table 6).

### DEGs of White- and Black-Seeded Varieties

**FIGURE 3:**
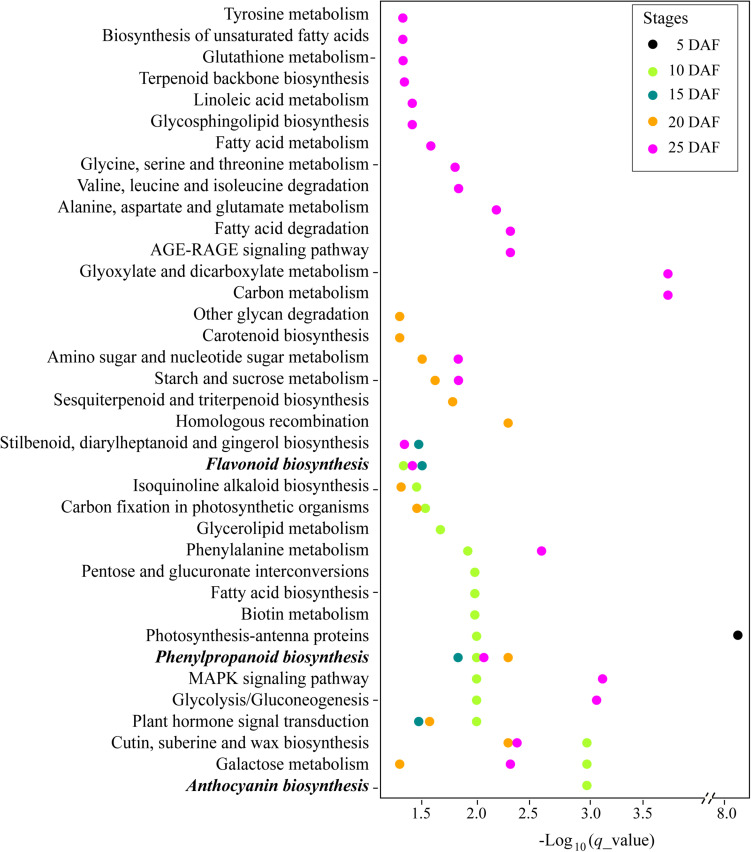
Kyoto Encyclopedia of Genes and Genomes (KEGG) enrichment analysis of the differentially expressed genes (DEGs) identified at the stages 5, 10, 15, 20, and 25 DAF between JS012 and Yuzhi 11.

To further explore the characteristics of the genes related with the seed coat color trait, we compared the expression profiles of the developing seeds in a white variety (Yuzhi 11) and a black variety (JS012) (Supplementary Figure 2). A total of 1,210.93 million reads (181.64 Gbases), with an average of 43.25 million clean reads, for the samples were harvested, and the mapping rates to the reference were all above 96.03% (Supplementary Table 7). The number of DEGs at the stages 5, 10, 15, 20, and 25 DAF between the two varieties were 2,148 (838 up and 1,310 down expressed in JS012), 5,176 (3,019 up and 2,157 down), 3,725 (1,598 up and 2,127 down), 2,984 (1,092 up and 1,892 down), and 5,115 (2,115 up and 3,000 down), respectively (Supplementary Table 8). GO annotation showed that the DEGs from the five stages presented a similar functional profile (Supplementary Figure 3). Most DEGs were involved in metabolic and cellular biological processes and the molecular functions of catalytic activity and binding. We further performed the KEGG enrichment analysis and found that the DEGs were significantly enriched in 37 pathways (*q*_value < 0.05), including pigment biosynthesis related phenylpropanoid biosynthesis (KEGG: ko00940, at the stages 10, 15, 20, and 25 DAF), flavonoid biosynthesis (KEGG: ko00941, at the stages 10, 15, and 25 DAF), and anthocyanin Biosynthesis (KEGG: ko00942, at the stage 10 DAF) pathways (Supplementary Table 9 and Figure 3). Notably, the three pigment biosynthesis related pathways above were enriched after the stage 5 DAF, and no genes in the three pathways were located in our *qSC* intervals (Supplementary Table 9).

### Seed Coat Color Candidate Genes Identification in White- and Black-Seeded Varieties

**FIGURE 4:**
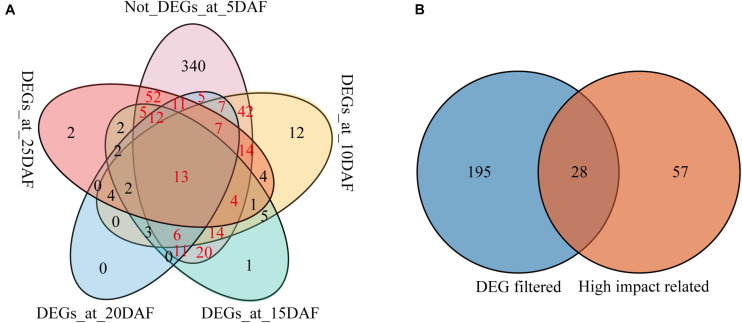
Venn diagram of genes in the *qSC* intervals. **(A)** Venn diagram of the DEGs after 5 DAF and not-DEGs at the stage 5 DAF; **(B)** Venn diagram of the genes from DEG filtered (the genes that were DEGs in at least one of the stages 10, 15, 20 and 25 DAF but not at 5 DAF), and high-impact related (the genes containing high-impacts variants and co-segregated with the seed coat color trait).

In order to screen the candidate genes related with the seed coat color regulation in sesame, we compared the expression profiles of the 625 genes in the *qSC* intervals during the seed development in the two varieties (Supplementary Table 10). Of the 625 genes, 62, 138, 101, 83, and 135 were differentially expressed at the stages 5, 10, 15, 20, and 25 DAF, respectively (Supplementary Table 10). Considering that the pigment was accumulated in JS012 gradually from 8 DAF and that no enrichment of the seed coat color formation related pathways were observed at the stage 5 DAF, we took the stage 5 DAF as the control and the other stages as the case to search for the candidate genes, and a Venn diagram consisting of the genes that were DEGs at the stages 10, 15, 20, and 25 DAF and not-DEGs at the stage 5 DAF was created (Figure 4A). We found 223 genes (the sum of the red numbers in Figure 4) that were DEGs in at least at one of the stages 10, 15, 20, and 25 DAF but not at 5 DAF. By selecting the intersection of the 223 genes and the 84 genes containing high-impacts variants (and co-segregated with the seed coat color trait; Supplementary Table 6), 28 genes were identified (Figure 4B).

The 28 genes were selected as the highly-potential candidate genes for the sesame seed coat color. Of the 28 candidates, seven were located on LG3, 2 on LG5, and 19 on LG9, while no genes were found on LG6 (Table 4). The 28 genes had various functions, including E3 ubiquitin-protein ligase (*C_2_3.773* and *C_2_3.787*), oxidase (*C_2_7.207* and *C34.291*), transcription factor (*C_2_3.819* and *C34.285*), and so on (Table 4). Of the 28 genes, 14 were expressed predominantly in JS012 (Supplementary Figure 4A), 11 expressed predominantly in Yuzhi 11 (Supplementary Figure 4B), and the other three genes presented the hybridized expression pattern (Supplementary Figure 4C).

**TABLE 4:** The 28 candidate genes related to seed coat color in sesame.

**LG**	**Gene**	**Annotated function**	**Related references^1^**
3	*C34.269*	Cyclic nucleotide-gated ion channel 1-like	
	*C34.277*	TRAFAC class dynamin-related protein 5A-like	
	*C34.281*	Protein aspartic protease in guard cell 2-like	
	*C34.285*	Transcription factor bHLH13-like isoform X1	Nesi et al., 2000; Nesi et al., 2001
	*C34.289*	Uncharacterized protein At4g00950-like	
	*C34.291*	Pyridoxine/pyridoxamine 5′-phosphate oxidase 2	Sang et al., 2011; Frank et al., 1999
	*C34.308*	NAC domain-containing protein 43-like	
5	*C_2_7.207*	Polyphenol oxidase I	Wei et al., 2015
	*C_2_7.220*	Hyoscyamine 6-dioxygenase	Polturak and Aharoni, 2018
9	*C_2_3.640*	Uncharacterized protein C1F8.04c-like	
	*C_2_3.644*	Serine/threonine-protein kinase CDL1-like	
	*C_2_3.683*	Xyloglucan endotransglucosylase/hydrolase protein 5	Wang et al., 2019
	*C_2_3.684*	Uncharacterized protein LOC105168501	
	*C_2_3.743*	Late blight resistance protein homolog R1A-4	
	*C_2_3.763*	Short-chain dehydrogenase TIC 32	
	*C_2_3.765*	Cell division cycle protein 48 homolog	
	*C_2_3.766*	Phosphatidylinositol transfer protein CSR1-like isoform X2	
	*C_2_3.767*	Purine permease 3-like	
	*C_2_3.768*	Deoxycytidylate deaminase	
	*C_2_3.771*	Uncharacterized protein LOC105179396	
	*C_2_3.773*	E3 ubiquitin-protein ligase BOI-like isoform X1	Tang et al., 2015
	*C_2_3.774*	Triose phosphate/phosphate translocator	Hilgers et al., 2018
	*C_2_3.780*	Histidine biosynthesis bifunctional protein hisIE, chloroplastic	
	*C_2_3.782*	Uncharacterized protein LOC105179408	
	*C_2_3.787*	E3 ubiquitin-protein ligase SIS3-like	Tang et al., 2015
	*C_2_3.819*	Transcription factor MYB1R1-like	Nesi et al., 2000; Nesi et al., 2001
	*C_2_3.850*	Cytochrome P450 94A2-like	Tanaka and Brugliera, 2013
	*C3.671*	Receptor-like protein kinase	

*^1^In these references, the corresponding genes had been proved or suggested to be involved in pigments biosynthesis or accumulation during seed formation.*

## Discussion

### The Multiple-Fold Improvement of the Sesame Genetic Linkage Map

In this study, a superdense genetic linkage map was constructed for sesame by genome resequencing and an updated map construction pipeline. Compared with the published genetic maps (Zhang Y. et al., 2013; Zhang et al., 2018; Wu et al., 2014; Uncu et al., 2016; Wang L. et al., 2016; Mei et al., 2017; Du et al., 2019; Asekova et al., 2021; Liang et al., 2021), the new genetic linkage map contains the highest number of both SNP/InDel markers (380,544) and bins (22,375) for sesame to date.

During the genetic map construction, the genotype data which resulted from genome resequencing usually have many noisy genotypes and always lead to inaccurate co-segregating marker (bins) grouping. To overcome the problem above, we developed a genetic map construction pipeline in 2016 and updated the method in this study. The previous pipeline basically consists of five steps: (1) calling variants individually; (2) choosing the variant loci that were called in either parent and in at least 20 progenies; (3) filtering out the loci that were heterozygous in either parent or that have a read depth under five in any progeny; (4) genotype coding; and (5) genotype correction (Zhang et al., 2016). In this study, the pipeline was improved, and the genotype matrix for map construction was prepared as follows: (1) calling variants jointly; (2) performing genotype coding; (3) combining the consecutive same markers within a short region; (4) genotype correction; and (5) filtering out the loci of distorted segregation. In the updated pipeline, joint variant calling was applied with a higher efficiency than the previous single-sample variant calling and loci filtration strategy. Joint variant calling shared the information across all samples in a cohort and therefore had greater sensitivity, particularly for the site with a low sequence coverage or misalignment (Van der Auwera et al., 2013). As to the loci filtration strategy, in order to “rescue” the deleted but informative sites, we just filtered out the loci with distorted segregation and omitted the filtration step before genotype correction. Thus, more makers and bins could be applied for map construction. In the study, the located SNP/InDel makers increased from 30,193 to 380,544 in the genetic map, with more than an 11-fold increase. The number of bins in the new map increased from 3,041 to 22,375, with more than a six-fold increase. Furthermore, to improve the accuracy of the pipeline, we had two times of merging involved in the pipeline, i.e., combining the consecutive and redundant markers within a short region into a representative marker, and grouping the adjacent and redundant markers into bins. The involvement of merging twice lead to an average of 17 markers per bin in the new map, and, thus, the new map should be of a greater accuracy.

### The Superdense Genetic Map Had More Robustly Dissected the Genetic Mechanism Underlying the Seed Coat Color Trait

Seed coat color is one of the earliest traits studied in crops for genetics analysis and Mendel’s law discovery. In sesame, seed coat color seems to be correlated with the species evolution and the beneficial effects on seed nutrition (Namiki, 2007; Zhang et al., 2012). Based on our investigation, the sesame seed coat color is stable under various environments, and is mainly influenced by the seed ripening level. With the aid of a molecular genetic map, Zhang H. et al. (2013) detected four QTLs for the seed coat color on three LGs using an F_2_ population derived from a cross between a white-seeded parent and a black-seeded parent. Wei et al. (2015) identified seven loci on three LGs for the trait by GWAS using a natural population. Wang L. et al. (2016) identified four QTLs on three LGs for the trait using a population consisting of 430 RILs. Lately, Du et al. (2019) found seven seed coat color related QTLs on three LGs. The results suggest that the seed coat color trait is controlled by a few major QTLs in sesame, and our phenotype analysis also agrees with this. In the present study, 17 QTLs in seven non-overlapping intervals on four LGs were determined for sesame coat color. Comparison of the results indicated that our QTLs covered all the previous published QTLs, except two loci, i.e., *qSCL-8.1* identified by Wang L. et al. (2016) and *qsccZ12* identified by Du et al. (2019). High consistency reflects the reliability of the QTL mapping results in this study. Furthermore, we found that the specific QTL *qSCL-8.1* reported by Wang L. et al. (2016) was close to the *qSC* cluster in this study. We proposed that *qSCL-8.1* should be another member of the cluster. The QTL *qsccZ12* was identified based on the color space index Z, which is different from the indices used in this study, and had the lowest phenotypic variation (5.58%) among the seven QTLs reported by Du et al. (2019). This may be the reason why we missed it in this study. Comparing with these previous studies, we (1) identified a new QTL for the seed coat color, i.e., *qSCa6.1*, and (2) successfully distinguished more QTLs than the previous studies.

### Candidate Genes for the Seed Coat Color in Sesame

In this study, in order to clarify the expression profiles of the candidate genes above, we also analyzed and compared the seed transcriptome during the seed development in the white- and black-seeded varieties. Compared to the stage 5 DAF, the DEGs at the following developing stages seem more important for the sesame seed coat color formation. Previous investigation indicated that pigment accumulation was usually observed approximately from 8 DAF in the black variety JS012 (data not shown). In this study, we determined that the pigment formation related pathways were not enriched at the time point 5 DAF. We thus used the DEGs at the stage 5 DAF to filter the other stages for candidate gene identification. Similarly, Wang et al. (2020) did a detailed research using RNA-seq data from a black- and white-sesame, and proposed that the genes controlling the sesame seed coat color were mainly expressed after 5 DAF, and thus took the 5 DAF as the initiated control and grouped 11–20 DAF to identify the candidate genes linked with pigment biosynthesis.

By integrating the QTL mapping and transcriptome analysis, we determined 28 genes as the candidate genes for the seed coat color in sesame (Table 4). Of the 28 candidate genes, the gene *SiPPO* (*C_2_7.207*) has been proved to determine the seed coat color in sesame (Wei et al., 2015), while the genes *C_2_3.819* and *C34.285* were annotated as transcription factor MYB-like and bHLH-like, respectively (Table 4). MYB and bHLH domain proteins act as a key regulator in PA accumulation in a developing seed (Nesi et al., 2000, 2001). Meanwhile, the gene *C34.291* was annotated to encode a pyridoxine/pyridoxamine 5′-phosphate oxidase (*PPOX*) gene, which was suggested to be related to pigment biosynthesis in both plant (Sang et al., 2011) and human (Frank et al., 1999). The gene *C_2_7.220* encodes a dioxygenase gene that was proven to be involved in plant pigments biosynthesis (Polturak and Aharoni, 2018). The gene *C_2_3.683* encodes a xyloglucan endotransglucosylase/hydrolase 5 (*XTH5*) genes, and *XTH5* has been reported to be involved in fruit pigment accumulation in the ripening processes in tomato (Wang et al., 2019). In addition, we found that the genes *C_2_3.773* and *C_2_3.787* were annotated as E3 ubiquitin-protein ligases, which were suggested to play important roles in tomato pigment accumulation (Tang et al., 2015). The genes *C_2_3.774* and *C_2_3.850* encode triose phosphate/phosphate translocator and cytochrome P450 gene, respectively, and were also suggested to be related to pigments biosynthesis in many plants (Tanaka and Brugliera, 2013; Hilgers et al., 2018). To sum up, 10 out of the 28 candidate genes were proved or suggested to be involved in pigments biosynthesis or accumulation. The results exhibited the high confidence of the candidate gene detection. However, we should remember that some SNP and InDels existed in the upstream and downstream sequences of the genes that have been omitted in the study. In the future, the function of the candidate genes and other genes in the target intervals should be further validated to clarify the regulation for the seed coat color trait in sesame.

## Data Availability Statement

The datasets presented in this study can be found in online repositories. The names of the repository/repositories and accession number(s) can be found below: https://www.ncbi.nlm.nih.gov/, PRJNA315474 and PRJNA742439.

## Author Contributions

CL performed the data analysis and drafted the manuscript. YD conducted the main experiments and participated in the data analysis. HM guided the experiments and drafted the manuscript. MJ conducted the cross-section. LW performed the genetic experiments. HZ conceived and designed the experiments, and guided manuscript correction for publishing. All authors have seen and approved the final version of the manuscript.

## Conflict of Interest

The authors declare that the research was conducted in the absence of any commercial or financial relationships that could be construed as a potential conflict of interest.

## Publisher’s Note

All claims expressed in this article are solely those of the authors and do not necessarily represent those of their affiliated organizations, or those of the publisher, the editors and the reviewers. Any product that may be evaluated in this article, or claim that may be made by its manufacturer, is not guaranteed or endorsed by the publisher.

## Supplementary Material

Supplementary Figure 1 Phenotypes of seed coat color of the two parents, F_1_ and some F_2_ progenies. **(A)** Yuzhi DS899; **(B)** JS012; **(C)** F_1_ seeds of the cross between Yuzhi DS899 and JS012; **(D)** seeds of some F_2_ progenies.

Supplementary Figure 2 The heatmap of the samples used for DEG screening between white- and black-seeded varieties.

Supplementary Figure 3 GO annotation of the DEGs identified between JS012 and Yuzhi 11 at the stages 5, 10, 15, 20, and 25 DAF, respectively.

Supplementary Figure 4 The expression pattern of the 28 candidate genes. The X-axis indicates the time points at 5, 10, 15, 20, and 25 DAF, respectively; the Y-axis indicates the expression level of the genes; ^∗^Indicates significantly differentially expressed; the orange line stands for the gene expression level in JS012, while the blue line stands for in Yuzhi 11. **(A)** the genes expressed predominantly in JS012; **(B)** the genes expressed predominantly in Yuzhi 11; **(C)** the genes with hybridized expression pattern.

Supplementary Table 1 Statistics of the *L*, *a*, and *b* values of the seed coat color trait in the F_2_ population derived from the cross between Yuzhi DS899 and JS012.

Supplementary Table 2 The variant counts of the two parents and their F_2_ population harvested from joint variant calling.

Supplementary Table 3 Distribution of bin markers in the superdense genetic map.

Supplementary Table 4 QTL mapping of seed coat color traits in sesame using the GCIM, ICIM, IM, and CIM methods.

Supplementary Table 5 The genes located in the *qSC* intervals.

Supplementary Table 6 The SNPs or InDels in the six contig segments between Yuzhi DS899 and JS012.

Supplementary Table 7 Statistics of the RNA-seq datasets used in the study.

Supplementary Table 8 The DEGs identified between JS012 and Yuzhi 11 at the stages 5, 10, 15, 20, and 25 DAF, respectively.

Supplementary Table 9 The enriched KEGG pathways in the DEGs between JS012 and Yuzhi 11 at the stages 5, 10, 15, 20, and 25 DAF, respectively.

Supplementary Table 10 The DEGs in the *qSC* intervals.
