# Supplementary material for: Identification of Candidate Genes Regulating the Seed Coat Color Trait in Sesame (Sesamum indicum L.) Using an Integrated Approach of QTL Mapping and Transcriptome Analysis
Source: Front Genet. 2021 Aug 4;12:700469. doi: 10.3389/fgene.2021.700469 (PMC8371934; doi:10.3389/fgene.2021.700469)
Supplement: Supplementary Figure 1 — Phenotypes of seed coat color of the two parents, F1 and some F2 progenies. (A) Yuzhi DS899; (B) JS012; (C) F1 seeds of the cross between Yuzhi DS899 and JS012; (D) seeds of some F2 progenies. [file Presentation_1.PPTX]

## Slide 1
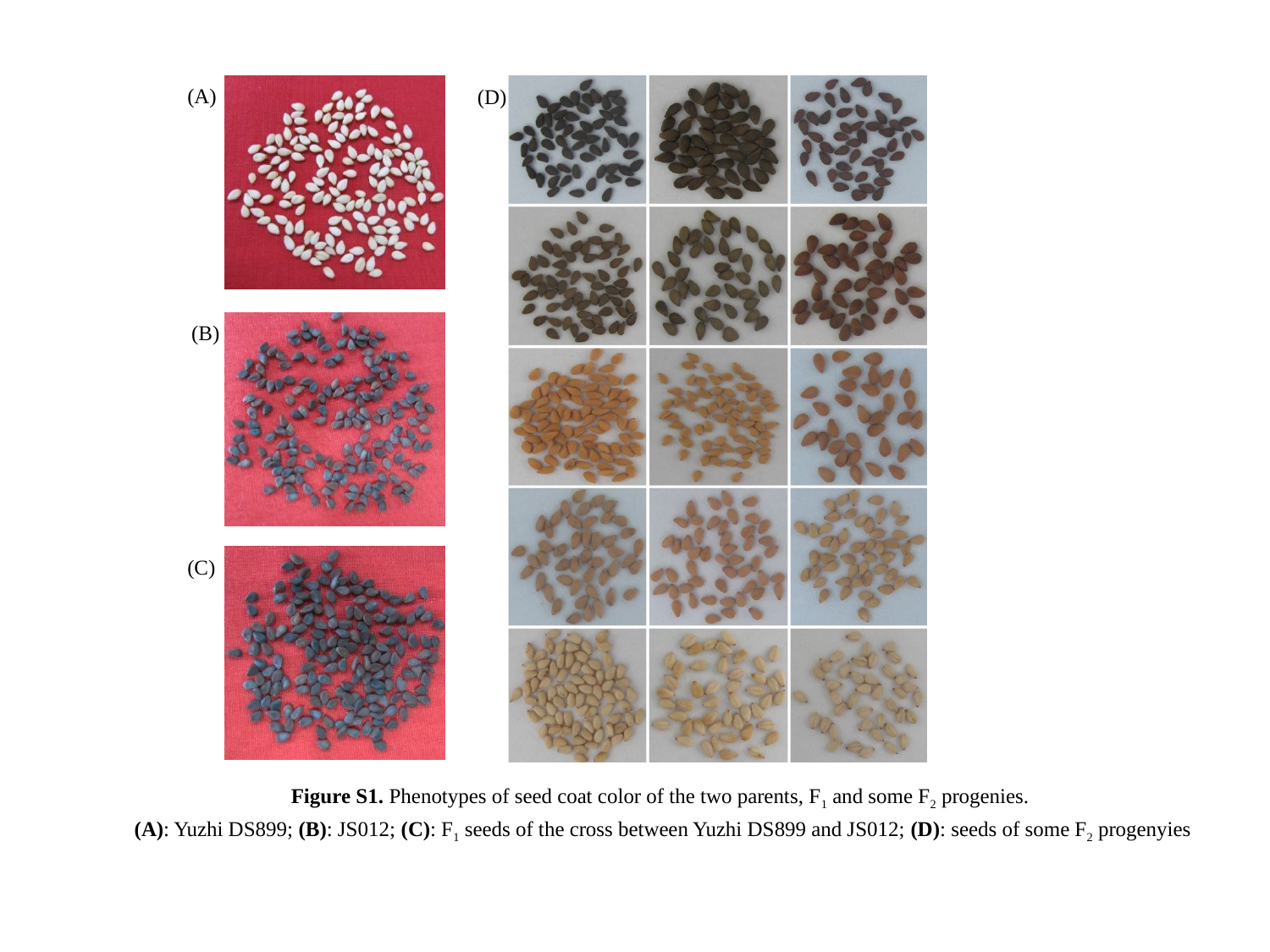

(A)
(D)
(B)
(C)
Figure S1. Phenotypes of seed coat color of the two parents, F1 and some F2 progenies.
 (A): Yuzhi DS899; (B): JS012; (C): F1 seeds of the cross between Yuzhi DS899 and JS012; (D): seeds of some F2 progenyies

## Slide 2
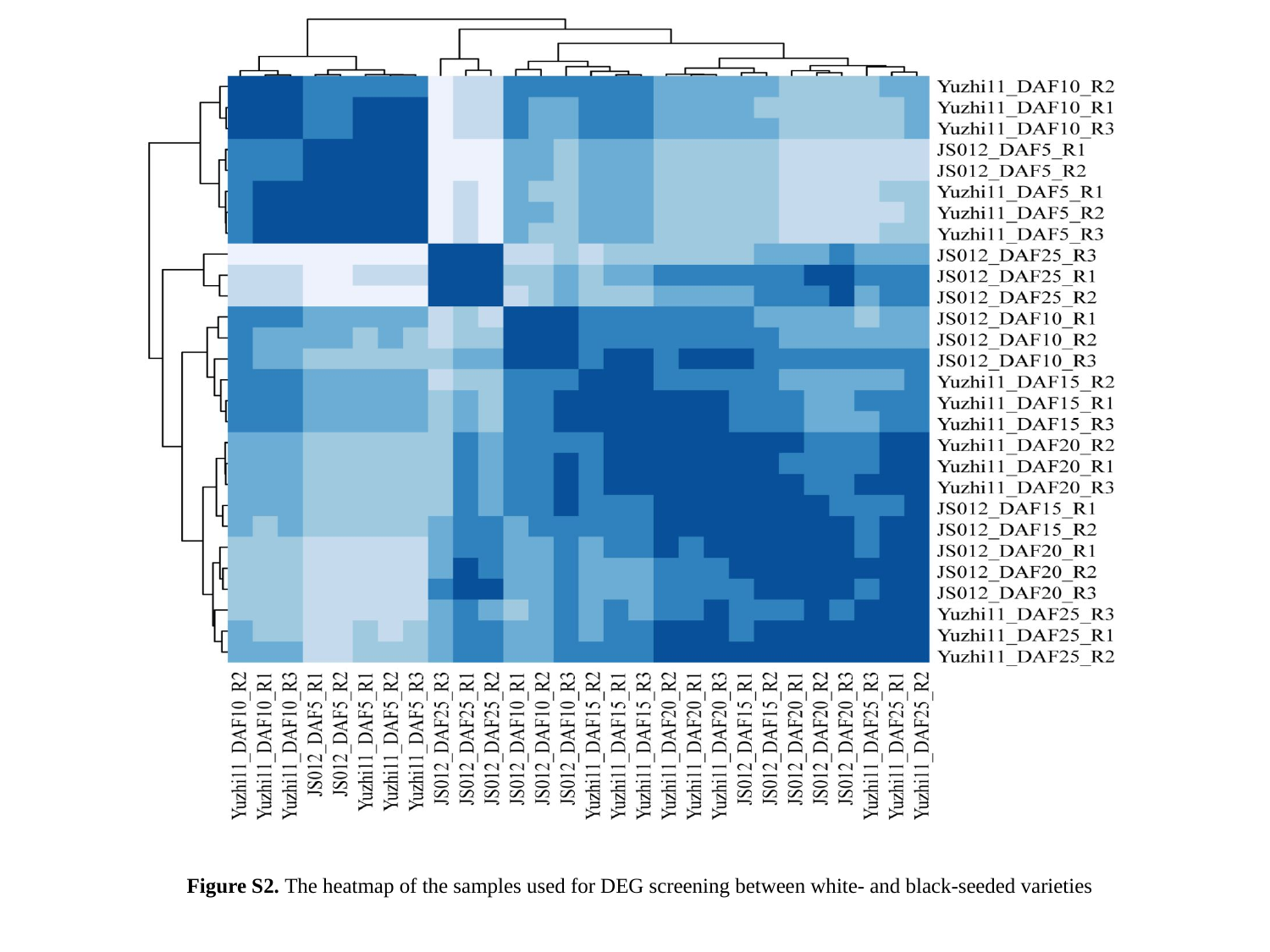

Figure S2. The heatmap of the samples used for DEG screening between white- and black-seeded varieties

## Slide 3
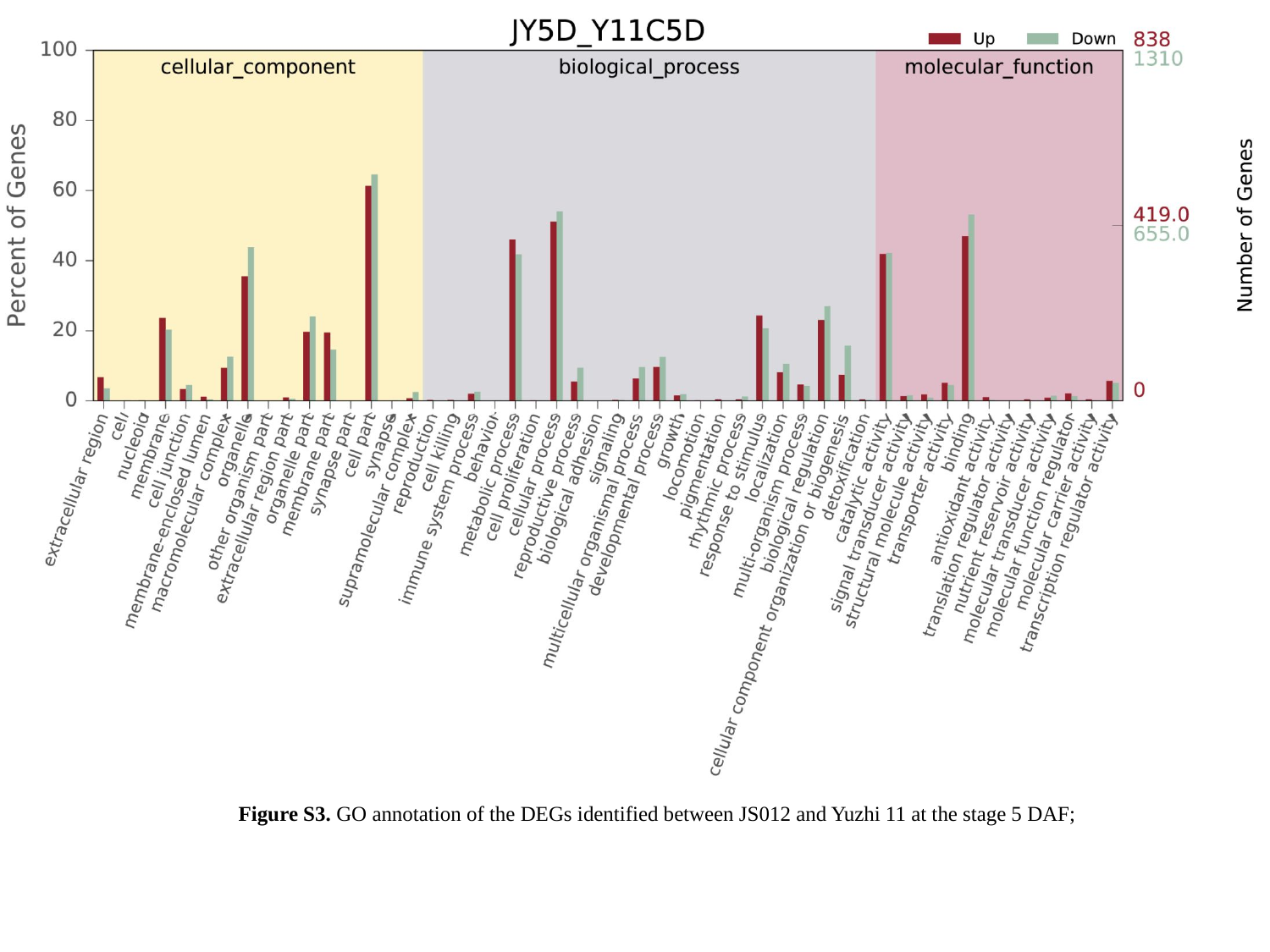

Figure S3. GO annotation of the DEGs identified between JS012 and Yuzhi 11 at the stage 5 DAF;

## Slide 4
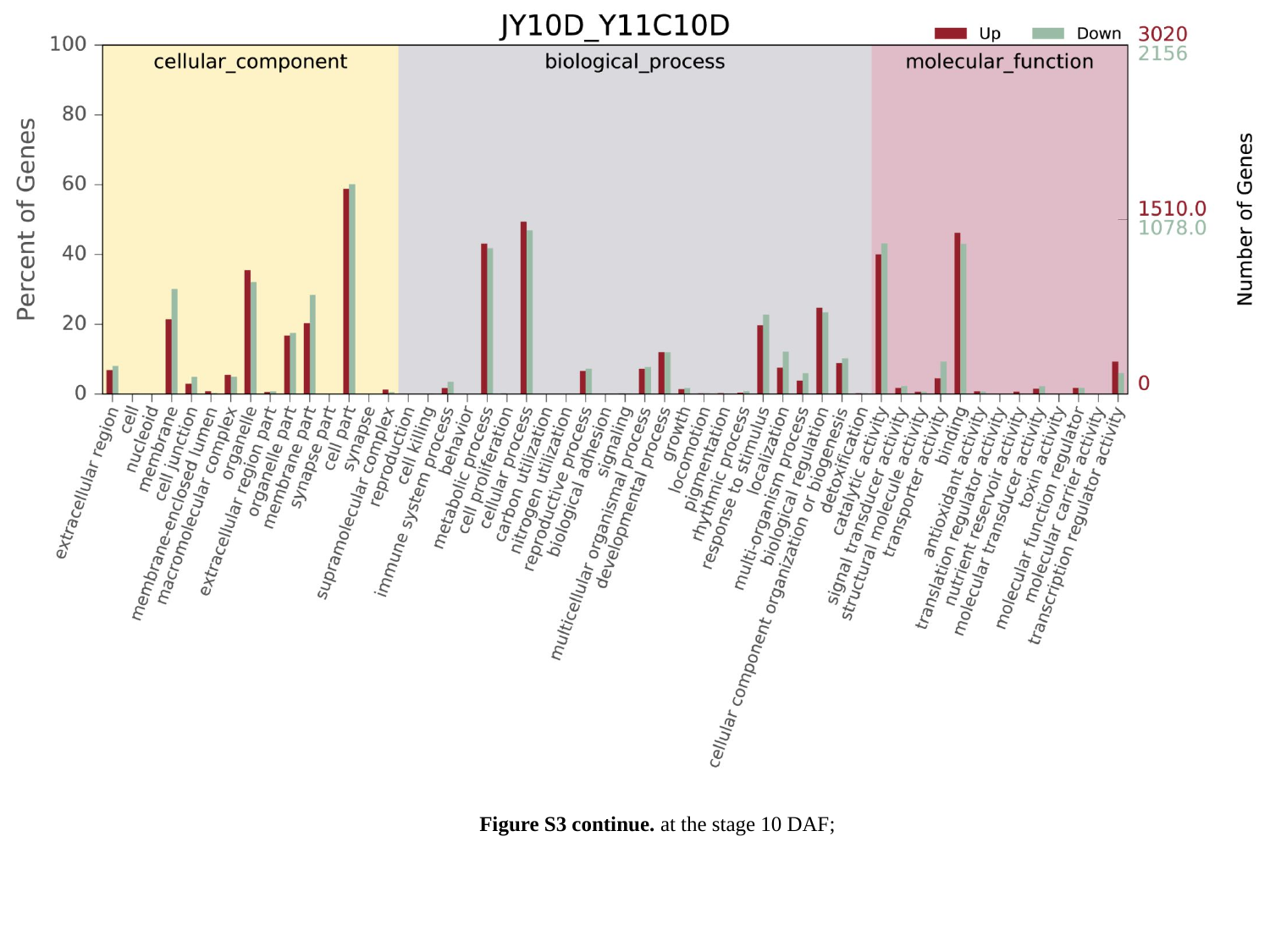

Figure S3 continue. at the stage 10 DAF;

## Slide 5
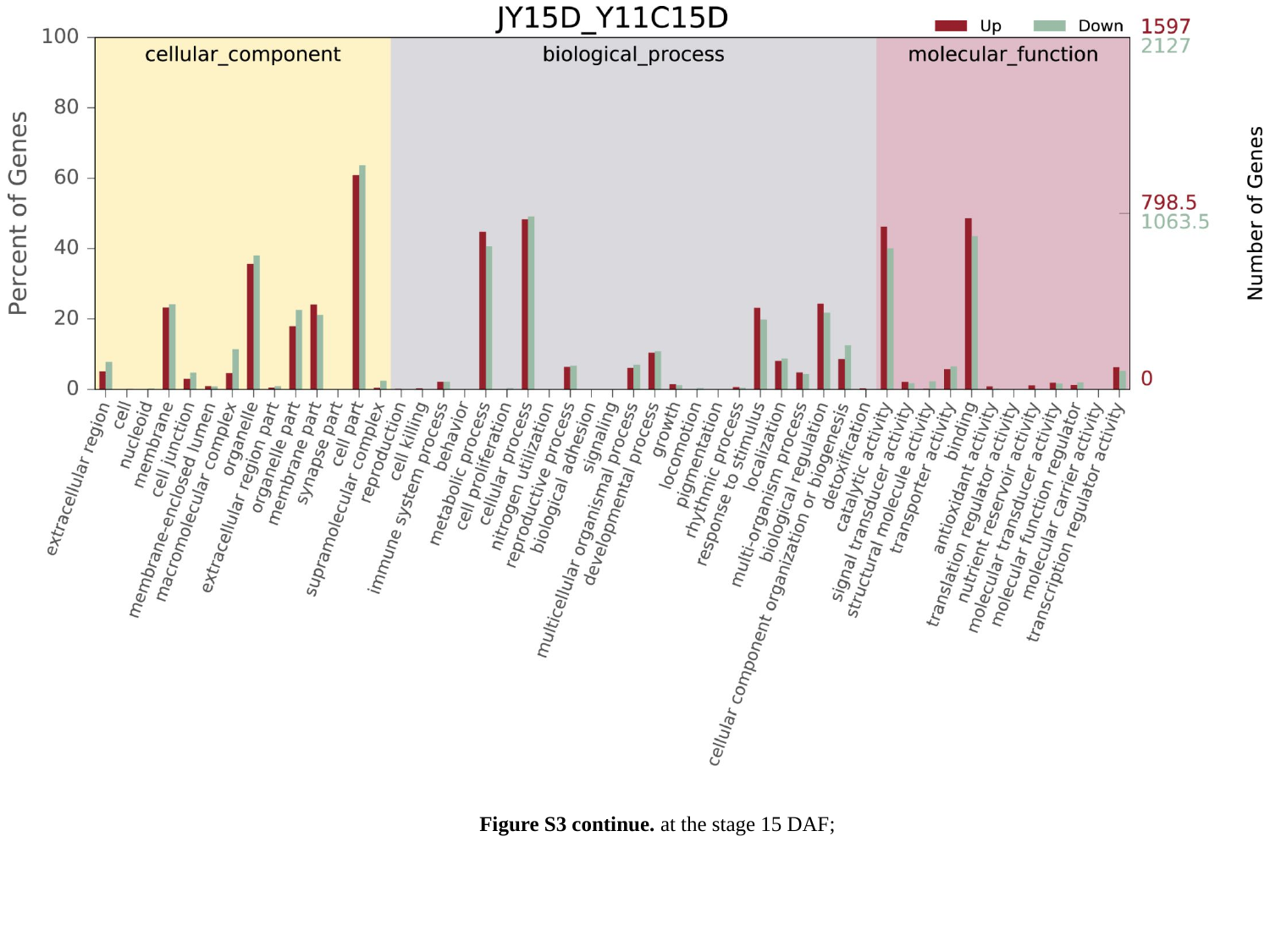

Figure S3 continue. at the stage 15 DAF;

## Slide 6
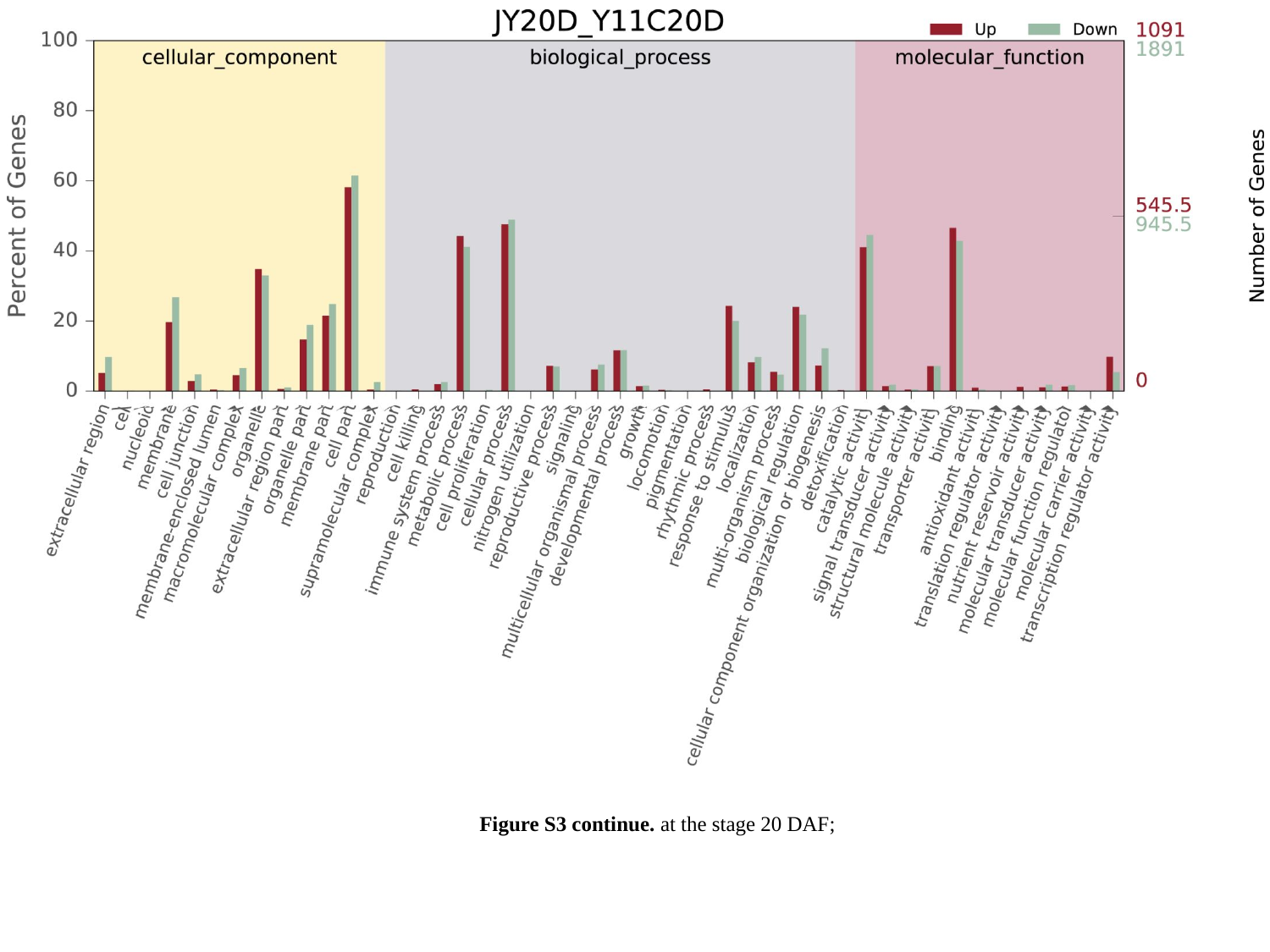

Figure S3 continue. at the stage 20 DAF;

## Slide 7
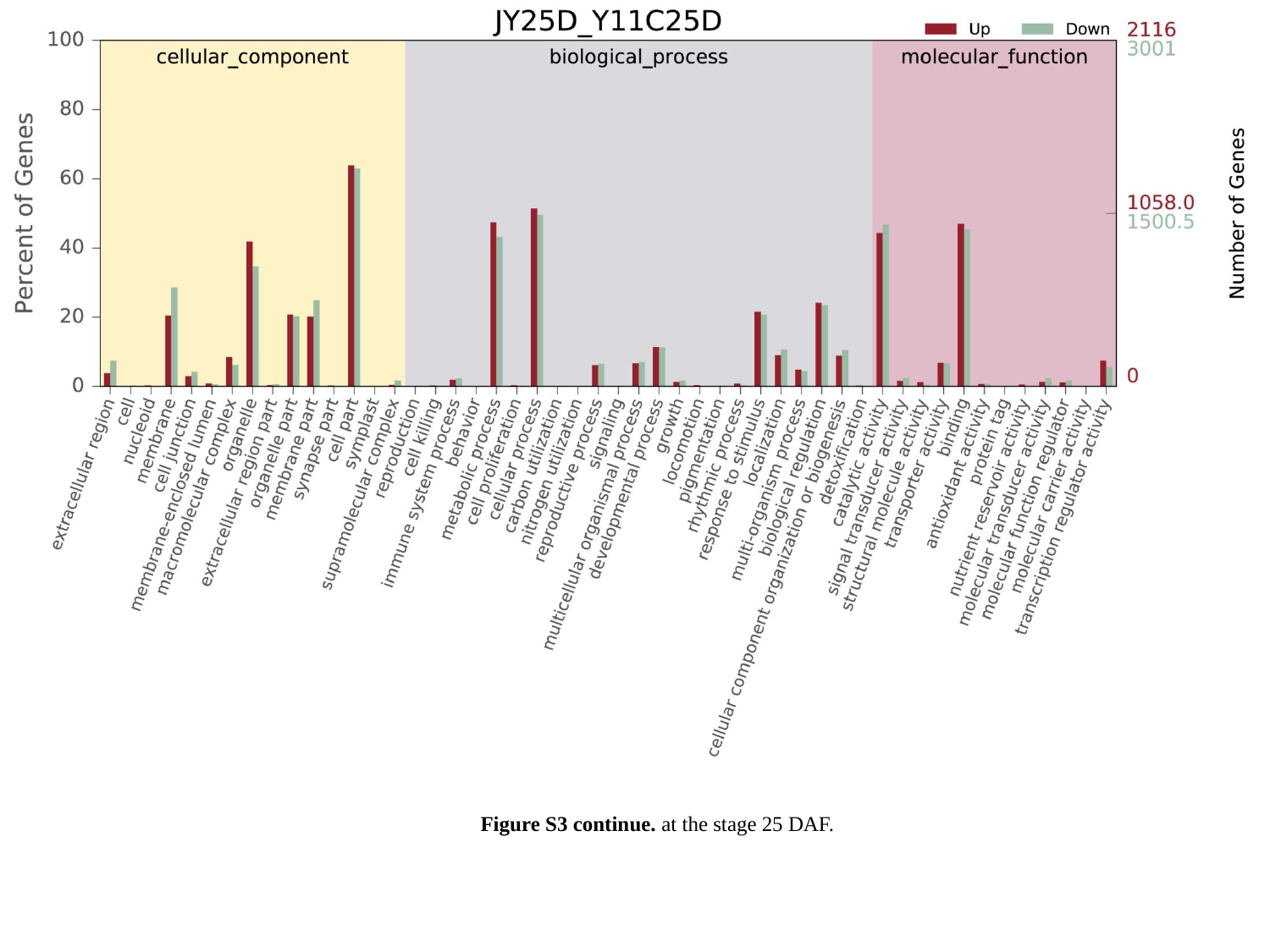

Figure S3 continue. at the stage 25 DAF.

## Slide 8
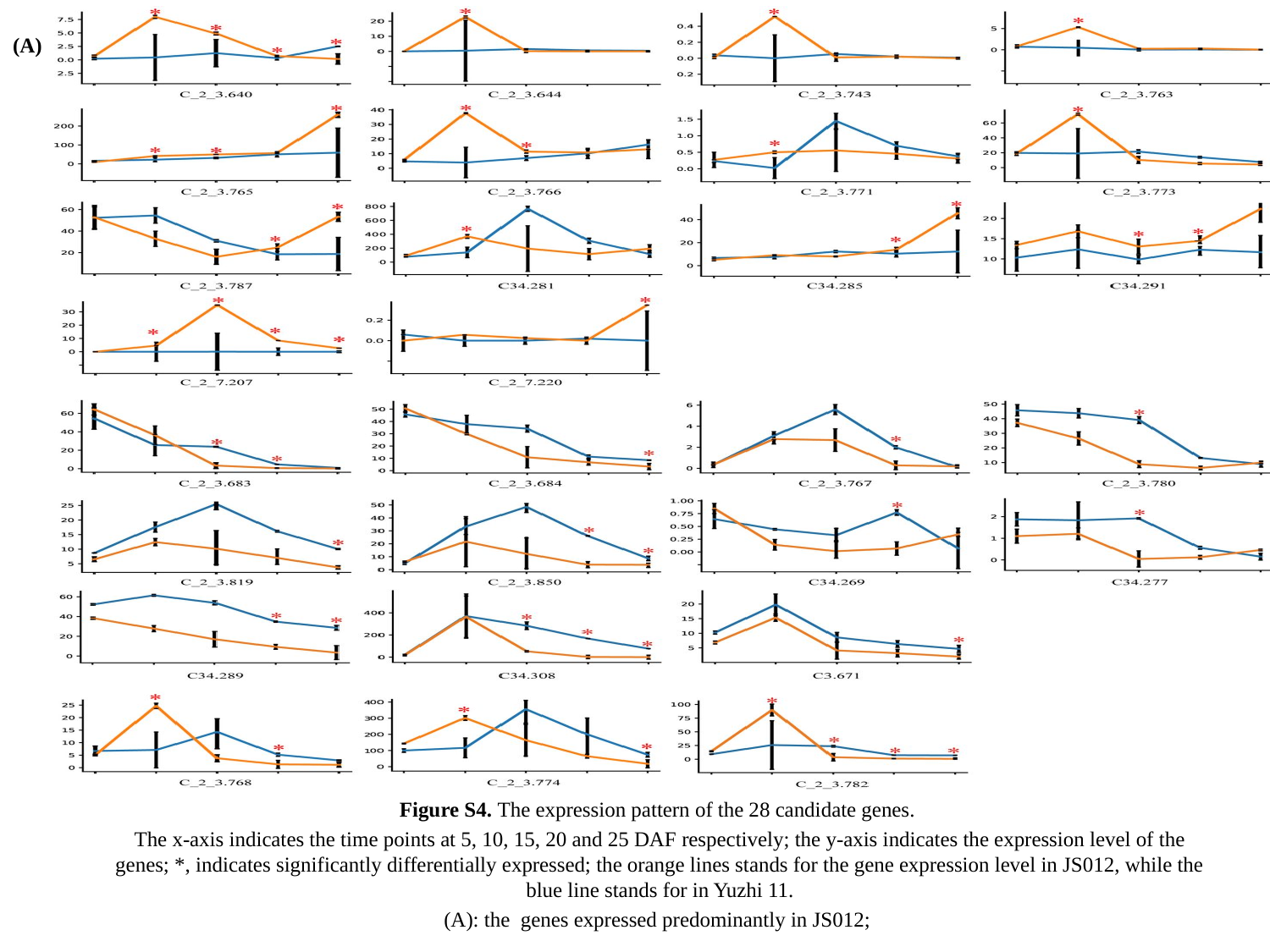

(A)
#
Figure S4. The expression pattern of the 28 candidate genes.
The x-axis indicates the time points at 5, 10, 15, 20 and 25 DAF respectively; the y-axis indicates the expression level of the genes; *, indicates significantly differentially expressed; the orange lines stands for the gene expression level in JS012, while the blue line stands for in Yuzhi 11.
(A): the genes expressed predominantly in JS012;

## Slide 9
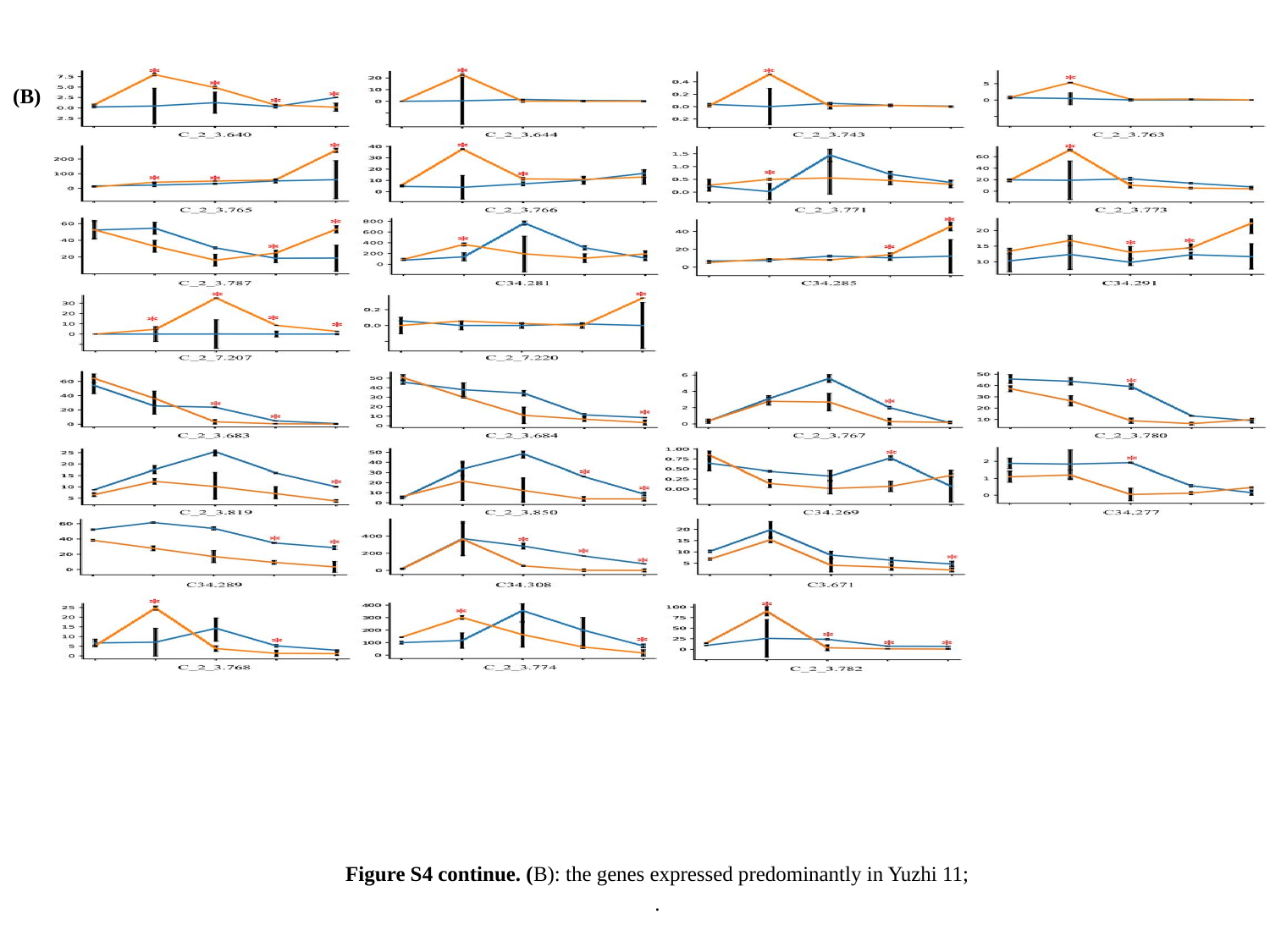

(B)
Figure S4 continue. (B): the genes expressed predominantly in Yuzhi 11;
.

## Slide 10
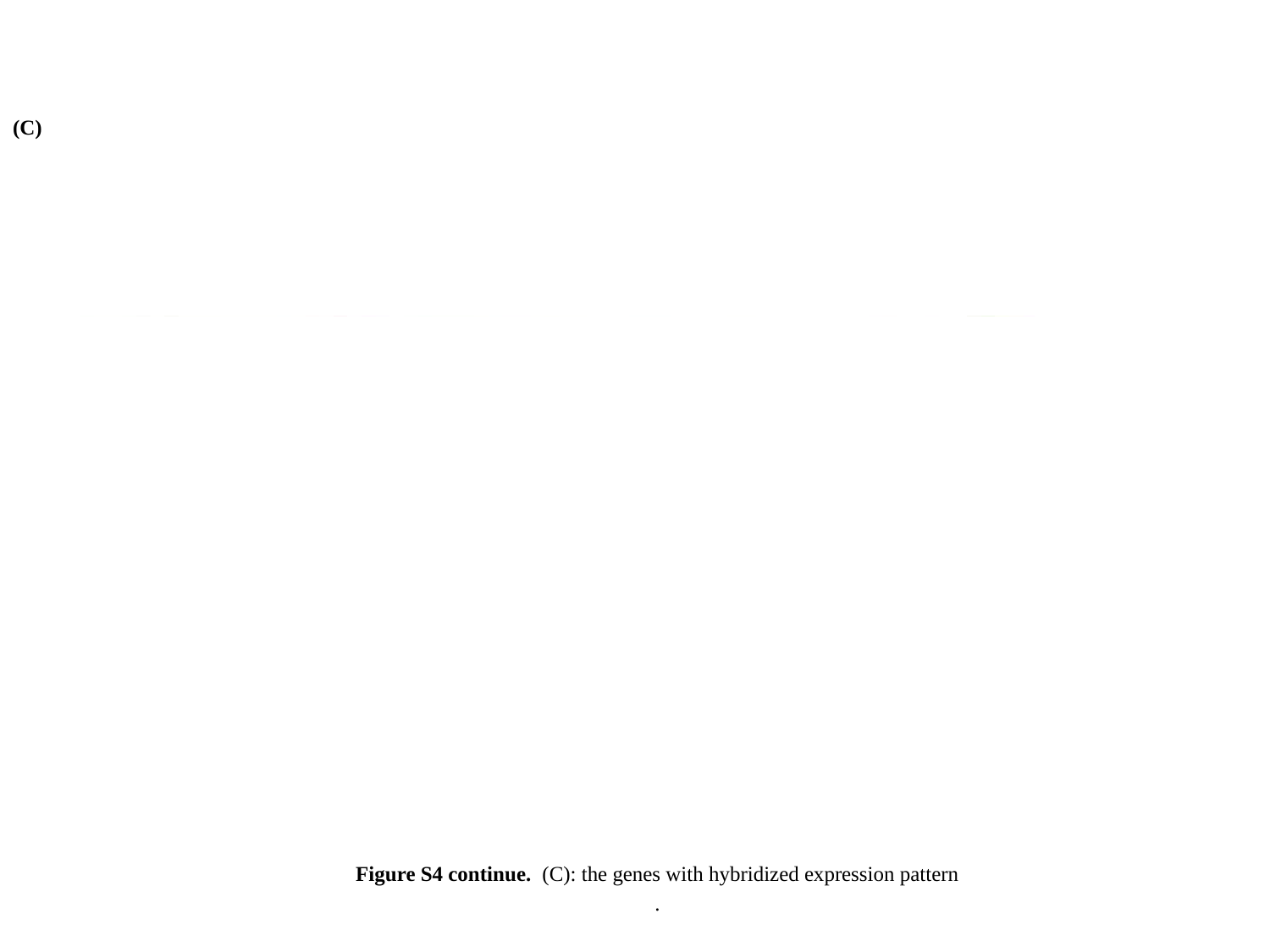

(C)
Figure S4 continue. (C): the genes with hybridized expression pattern
.
